# Meta-Analysis of the Association between Asbestos Exposure and Esophageal Cancer

**DOI:** 10.3390/ijerph182111088

**Published:** 2021-10-21

**Authors:** Chih-Wei Wu, Hung-Yi Chuang, Dong-Lin Tsai, Tzu-Yu Kuo, Chen-Cheng Yang, Huang-Chi Chen, Chao-Hung Kuo

**Affiliations:** 1 Department of Occupational and Environmental Medicine, Kaohsiung Municipal Siaogang Hospital, Kaohsiung Medical University, Kaohsiung City 812, Taiwan; M016170@ms.skh.org.tw or; 2 Department of Surgery, Shin Kong Wu Ho Su Memorial Hospital, Taipei City 111, Taiwan; 3 Department of Occupational and Environmental Medicine, Kaohsiung Medical University Hospital, Kaohsiung Medical University, Kaohsiung City 807, Taiwan; ericch@kmu.edu.tw; 4 Graduate Institute of Medicine, Kaohsiung Medical University, Kaohsiung City 812, Taiwan; 5 Division of Thoracic Surgery, Department of Surgery, Kaohsiung Municipal Siaogang Hospital, Kaohsiung Medical University, Kaohsiung City 812, Taiwan; anakin1112@hotmail.com; 6 Division of Pulmonary Medicine, Department of Internal Medicine, Kaohsiung Municipal Siaogang Hospital, Kaohsiung Medical University, Kaohsiung City 812, Taiwan; 980262@kmuh.org.tw; 7 Cancer Center, Kaohsiung Municipal Siaogang Hospital, Kaohsiung Medical University, Kaohsiung City 812, Taiwan; huangchichen@gmail.com; 8 Division of Gastroenterology, Department of Internal Medicine, Kaohsiung Municipal Siaogang Hospital, Kaohsiung Medical University, Kaohsiung City 812, Taiwan; jhkao@kmu.edu.tw

**Keywords:** asbestos exposure, esophageal cancer, carcinogen, occupational medicine, environmental medicine, meta-analysis

## Abstract

Background: We conducted a meta-analysis to quantitatively assess the association between asbestos exposure and esophageal cancer. Methods: We systematically collected articles from three electronic databases and calculated the pooled standardized mortality rate (SMR) from the meta-analysis. Subgroup analysis according to the type of asbestos exposure, follow-up years, sample size, industry classification, sex, and high-dose exposure was conducted. Results: From 242 studies, 34 cohort studies were included in our meta-analysis. Pooled SMR was positively associated with asbestos exposure and esophageal cancer (pooled SMR = 1.28; 95% confidence interval (CI) 1.19–1.38, p < 0.00001). In the subgroup analysis, (1) chrysolite, (2) four groups with follow-up over ten years, (3) the textile industry and shipyard, (4) both male and female, and (5) eight studies on highest asbestos exposure, all the subgroups showed significantly increased pooled SMRs. Conclusion: Asbestos exposure was significantly and positively associated with esophageal cancer, especially chrysolite. Considering the long latency period, we suggest that patients should be followed up for cancer, including esophageal cancer, for over ten years.

## 1. Introduction

Asbestos has been classified as a group 1 carcinogen (carcinogenic to humans) since the 1970s [1]. Exposure to asbestos may result in asbestosis, recurrent pleural or pericardial effusion, pleural plaque and malignancy, including pleural and peritoneal mesothelioma, pharyngeal cancer, laryngeal cancer and lung cancer [1,2].

After being inhaled or ingested into the body, longer asbestos fibers cannot be efficiently engulfed and cleared by macrophages [3]. The accumulation of asbestos fibers catalyzes the generation of free radicals and increases the uptake and metabolism of many specific proteins as well as carcinogenic molecules (for example, polycyclic aromatic hydrocarbons) by respiratory tract epithelial cells [4,5]. In addition, asbestos bodies formed by “frustrated phagocytosis” and surrounding inflammatory cells induce chronic inflammation of affected tissue [3]. The above mechanism results in the formation of mesothelioma and respiratory tract malignancies.

Esophageal cancer is a common cancer worldwide and is the sixth leading cause of cancer death, accounting for over 500,000 cancer deaths annually (approximately 5.3% of all global cancer deaths) [6]. Esophageal cancer can be classified into squamous cell carcinoma and adenocarcinoma based on histology. In addition, men are at three to four times higher risk of developing esophageal squamous cell carcinoma and seven to ten times higher risk of developing esophageal adenocarcinoma [6]. Risk factors for esophageal cancer include genetic factors, sex, race, gastroesophageal reflux disease, obesity, nitrosamine, tobacco, alcohol consumption, drug use, low socioeconomic status, and nutritional deficiency [6,7]. However, studies on occupational or environmental factors of esophageal cancer are still rare.

The causal link between asbestos and gastrointestinal cancer has been discussed since asbestos is regarded as a group 1 carcinogen and is associated with asbestos exposure. However, the association between asbestos exposure and esophageal cancer has been debated in the last 20–30 years. Morgan et al. demonstrated that asbestos exposure may elevate the risk of esophageal cancer (standardized mortality ratio (SMR): 2.38; 95% confidence interval (CI) 1.45–3.68) and total gastrointestinal cancer (SMR: 1.05; 95% CI 0.98–1.13) [8]. In a large cohort of 58,279 employees, Offermans et al. shown an increase hazard ratio (2.22, 95% CI 1.00–4.94) of esophageal cancer in the asbestos exposure workers [9]. In a study of 4427 shipbreaking workers, Wu et al. found an increased hazard ratio (2.31, 95% CI 1.00–5.41) of esophageal cancer in asbestos exposure [10]. Clin et al. analyzed 2024 subjects with history of occupational asbestos exposure and they found increased incidence of esophageal cancer (standardized incidence ratio (SIR) 1.60, 95% CI 1.00–2.42) [11]. On the other hand, Reid et al. estimated 129 cases with a history of occupational crocidolite (one classification of asbestos) exposure, and the results revealed no significantly association between asbestos exposure and esophageal cancer (SIR 1.11 with 95% CI 0.60~2.07, (SMR): 0.89 with 95% CI 0.44~1.78) [12]. Similar result was also found on cohort study of workers with history of asbestos exposure from de La Provote et al. (SIR 1.29, 95% CI 0.73–2.09) [13]. Moreover, Gustavsson et al. [14], Parent et al. [15], and Santibañez et al. [16] also shown no significantly increased risk of esophageal cancer in asbestos exposure case control studies, which revealed relative risk (RR) 1.21 (95% CI 0.67–2.17), odds ratio (OR) 1.4 (95% CI 0.7–2.7), and OR 1.27 (95% CI 0.77–2.10), respectively. The association between asbestos exposure and esophageal cancer was still inconsistent till now.

Due to the controversial and inconsistent relationship between asbestos exposure and esophageal cancer, our aim was to conduct a meta-analysis to investigate the association between asbestos exposure and esophageal cancer.

## 2. Materials and Methods

### 2.1. Protocol and Registration

We conducted a meta-analysis of the association between asbestos exposure and esophageal cancer based on the guidelines of Preferred Reporting Items for Systematic Reviews and Meta-Analyses (PRISMA). The review protocol is registered at the International Prospective Register of Systematic Reviews (PROSPERO) (ID: 265920, under the process of registration), which is an open-access online database of prospectively registered systematic reviews on topics related to health and social care.

### 2.2. Data Sources and Search Terms

We initially searched related studies in electronic databases, including PubMed, Embase and Web of Science, on May 16, 2021. We did not set any limitations on publication date, and all studies containing target keywords were identified. Initial searches of research using various keywords were performed by two researchers (H.Y. Chuang, and C.C. Yang). Keyword combinations proposed by researchers were as follows: “Asbestos” OR “Asbestos, Amphibole” OR “Asbestos, Amosite” OR “Asbestos, Crocidolite” OR “Asbestos, Serpentine” OR “Asbestos, Amphibole-group Minerals” OR “Asbestos, Amphibole group Minerals” OR “Amphibole Asbestos” OR “Amphiboles” OR “Amphibole” OR “Crocidolite” OR “Crocidolite Asbestos” OR “Blue Asbestos” OR “Asbestos, Blue” OR “Asbestos, Crocidolite” OR “Asbestos, Serpentine” OR “Asbestosis” AND “Esophageal Neoplasm” OR “Neoplasm, Esophageal” OR “Esophagus Neoplasm” OR “Esophagus Neoplasms” OR “Neoplasm, Esophagus” OR “Neoplasms, Esophagus” OR “Neoplasms, Esophageal” OR “Cancer of Esophagus” OR “Cancer of the Esophagus” OR “Esophagus Cancer” OR “Cancer, Esophagus” OR “Cancers, Esophagus” OR “Esophagus Cancers” OR “Esophageal Cancer” OR “Cancer, Esophageal” OR “Cancers, Esophageal” OR “Esophageal Cancers” OR “Esophageal Squamous Cell Carcinoma” OR “Esophageal Neoplasms”(Mesh) OR “Esophageal Neoplasms”. However, words such as actinolite, tremolite, and anthophyllite do not belong the entry terms about asbestos in PubMed database were not use in the search. We considered the search strategies for the Embase and Web of Science databases as appropriate.

### 2.3. Eligibility Criteria

Studies were included based on the following inclusion criteria: (1) no participant limitation, (2) history of asbestos exposure, and (3) outcome of esophageal cancer. (4) studies mentioning SMR, or mentioning observed and expected group. Studies were excluded according to the following exclusion criteria: (1) studies lacking key information about asbestos exposure and esophageal cancer, (2) studies without full-text or relevant data resources could not be obtained, (3) letters, reviews, case reports, expert opinions, or laboratory studies, (4) nonhuman research, (5) guidelines, (6) articles focusing on policy discussion, and (7) articles not included during analysis.

### 2.4. Study Selection Process

Initially, first-time screening was performed by two investigators (C.W. Wu and C.C. Yang) by assessing the titles and abstracts of preliminarily identified studies. A second round of screening was performed by screening the full text of articles meeting the eligibility criteria and those with unclear eligibility. Five researchers (H.Y. Chuang, D.L. Tsai, T.Y. Kuo, H.C. Chen, and C.H. Kuo) further comprehensively evaluated the eligibility of each study to consider whether it should be included if two researchers initially disagreed on the eligibility of the study.

### 2.5. Data Collection

From each article included in our study, information regarding study characteristics, asbestos exposure, and esophageal cancer was extracted. We also obtained the association between asbestos exposure and esophageal cancer. We contacted the corresponding authors for further verification if the above information was ambiguously described or was mentioned in doubt.

### 2.6. Study Characteristics

Data related to study characteristics were extracted as follows: first author, publication year, country where the study was completed, sample size, characteristics of participants, and number of outcome events (for example, the observed and expected number of participants with esophageal cancer, or the standardized mortality ratio (SMR)).

### 2.7. Asbestos Exposure

Asbestos exposure was defined based on individual studies, including past working history provided by factories, national agencies, or solitary institutions, questionnaires, and information from interviews. In addition, information on dust measurement and cumulative fiber dose was provided according to individual studies.

### 2.8. Esophageal Cancer

We defined esophageal cancer as one of the causes of death described in our included studies, which was validated by authors through death certificates, records provided by institutions or hospitals (including hospitalization records, reports from histological material or autopsy), and International Classification of Disease (International Statistical Classification of Disease, Injuries, and Cause of Death) codes.

### 2.9. Statistical Analysis

We calculated all SMRs from the number of observed deaths and patients with esophageal cancer, with the mortality rate for the population regarded as the basis for comparison, and their 95% CIs were recalculated according to the Boice-Monsom method [17]. We derived the pooled standardized mortality rate (SMR) from the respective SMR of each included study, and the standard error (SE) for the SMR was assessed according to the 95% confidence interval (95% CI). If SMR wasn not mentioned in articles, we recalculated SMR and its 95% CI through observed death and expected death mentioned in articles [18]. We used the main SMRs and SEs to estimate the pooled SMR and its 95% CI through a fixed-effects model while conducting the main analysis. We applied the fixed-effects model to assess the possibility of heterogeneity in SMRs among the studies included based on the study characteristics. We quantified the effect of the heterogeneity among the included studies by using I^2^ statistics. Publication bias was estimated by using a funnel plot. We performed further subgroup meta-analysis of the included studies including type of asbestos exposure, follow-up years of the included studies, sample size, industry classification, gender, and highest exposure group. All statistical analyses were conducted using Review Manager version 5.4 and R version 3.6.2.

## 3. Results

### 3.1. Selected Studies

**Figure 1:**
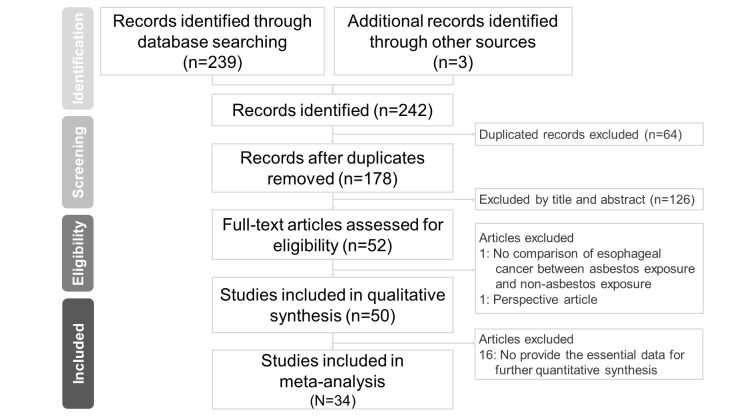
Preferred Reporting Items for Systematic Reviews and Meta-Analyses flow chart.

Figure 1 shows the PRISMA flow chart of the selection procedure. In the first step, we found 239 articles from three databases (PubMed, Embase, and Web of Science), and an additional three studies were identified by reference screening [19,20]. Among the 242 articles, 64 duplicates were removed, and two authors (C.W. Wu, and C.C. Yang) recognized the remaining 178 studies through title and abstract screening. After excluding 126 studies by title and abstract, two authors assessed the full-text articles of the 52 studies for eligibility. After screening the full text of the 52 articles, two studies were omitted because they did not meet the following criteria: no comparison of esophageal cancer between the asbestos exposure and reference populations (N = 1) and a perspective article (N = 1). Finally, 50 studies were included in the qualitative synthesis, and 34 studies were included in the quantitative synthesis using meta-analysis.

### 3.2. Study Characteristics

Table 1 demonstrates the basic characteristics of the 34 included studies [19,20,21,22,23,24,25,26,27,28,29,30,31,32,33,34,35,36,37,38,39,40,41,42,43,44,45,46,47,48,49,50,51,52]. Among the included studies, all 34 were cohort studies, of which the earliest was published in 1963, while the latest was published in 2017. Most of the studies were conducted in both genders or mainly in males, while four studies focused on females or performed subgroup analyses of females [34,40,50,52]. Different asbestos types, including seven studies of chrysolite [30,34,39,44,46,49,50], four studies of amosite [20,22,32,51], two studies of crocidolite [28,42], and 20 studies of mixed asbestos, were investigated for further subgroup analysis of the type of asbestos. Two studies had a follow up of less than or equal to 10 years [23,34,52], seven studies were above 10 years but no more than 20 years [19,20,27,29,30,32,50], six studies were above 20 years but no more than 30 years [38,39,42,46,48,49], eight studies were above 30 years but no more than 40 years [21,22,26,28,41,43,47,51], and eight studies were above 40 years [24,31,35,36,37,44,45,52]. The sample size of five studies was less than 1000 participants [20,32,38,45,47], 20 studies were between 1000 and 10,000 [19,22,23,24,26,27,28,31,33,34,37,39,40,42,44,46,48,49,51,52], and eight studies were over 10,000 [21,29,30,35,36,41,43,50]. Regarding industry classification, seven studies focused on the textile industry [23,33,34,39,40,44,47], three studies focused on the shipyard industry [36,45,48], and five studies focused on miners [28,30,42,46,49]. Eight studies included the highest asbestos exposure subgroup according to their individual definition [23,25,27,31,33,45,48,49].

### 3.3. Meta-Analysis

**Figure 2:**
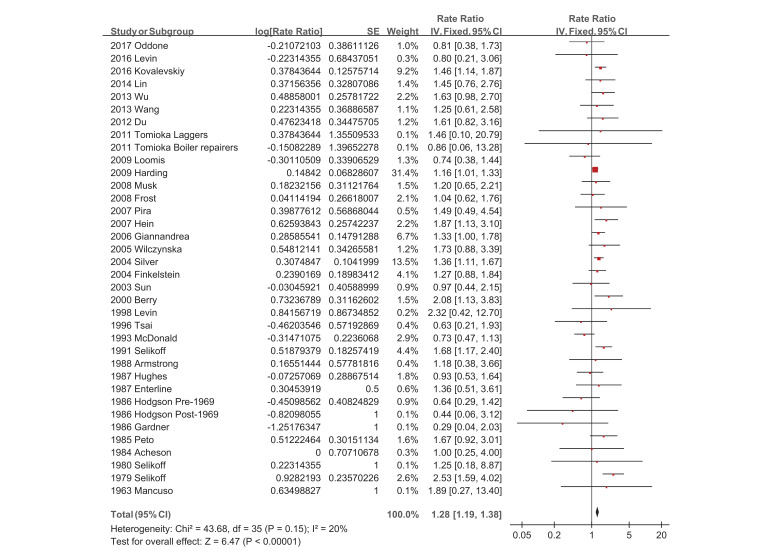
Asbestos exposure and standardized mortality ratio (SMRs) of esophageal cancer in the 33 studies: a fixed-effect model.

**Figure 3:**
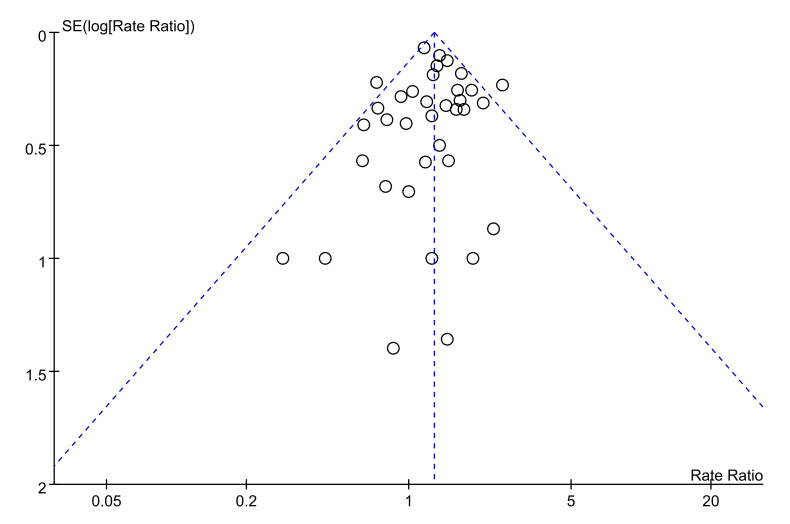
Funnel plot of log-transformed standardized mortality ratios (SMRs) of asbestos exposure and esophageal cancer and standard errors for the 33 studies.

The association between asbestos exposure and esophageal cancer was determined by a fixed-effect model meta-analysis, in which the pooled SMR resulted from 36 SMRs of 34 studies (Table 1, Figure 2) [19,20,21,22,23,24,25,26,27,28,29,30,31,32,33,34,35,36,37,38,39,40,41,42,43,44,45,46,47,48,49,50,51,52]. Compared with the reference group, the pooled SMR of esophageal cancer was significantly increased in participants with asbestos exposure (pooled SMR = 1.28; 95% CI 1.19–1.38; z = 6.47, *p* < 0.00001). The heterogeneity was not significant (I^2^ = 20%, χ^2^ = 43.68, *p* = 0.15). Figure 3 shows a funnel plot of the log transformed SMRs of the 34 studies, and the SEs revealed significant SMRs with relatively and reasonably smaller SEs.

### 3.4. Subgroup Analysis

**Figure 4:**
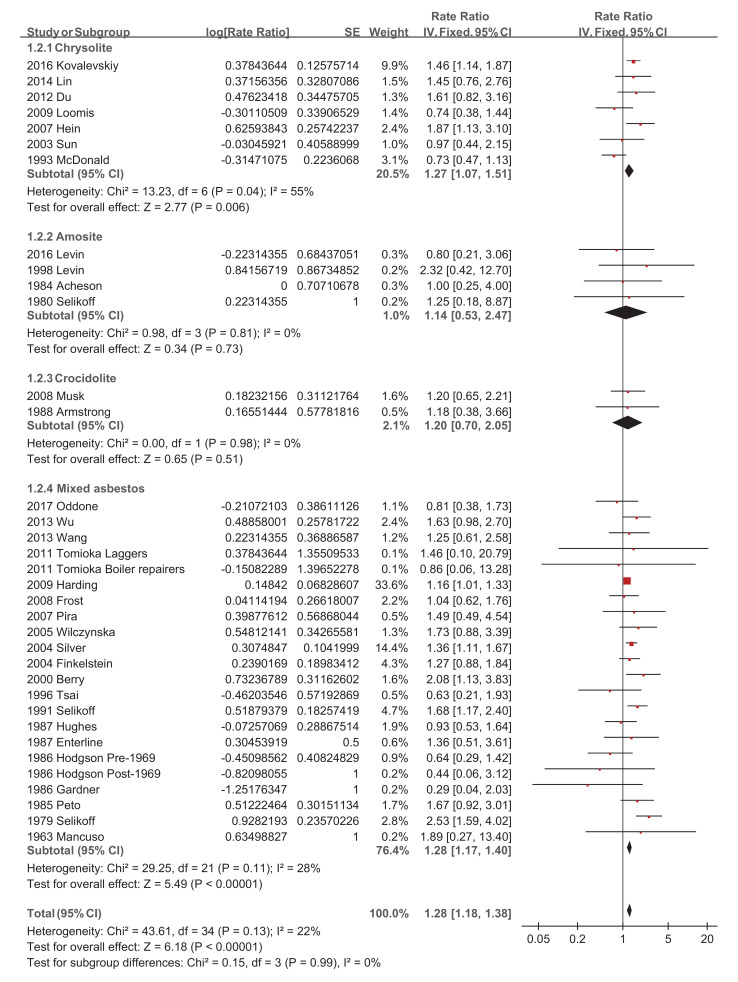
Subgroup analysis of SMRs of esophageal cancer based on the type of asbestos exposure.

**Figure 5:**
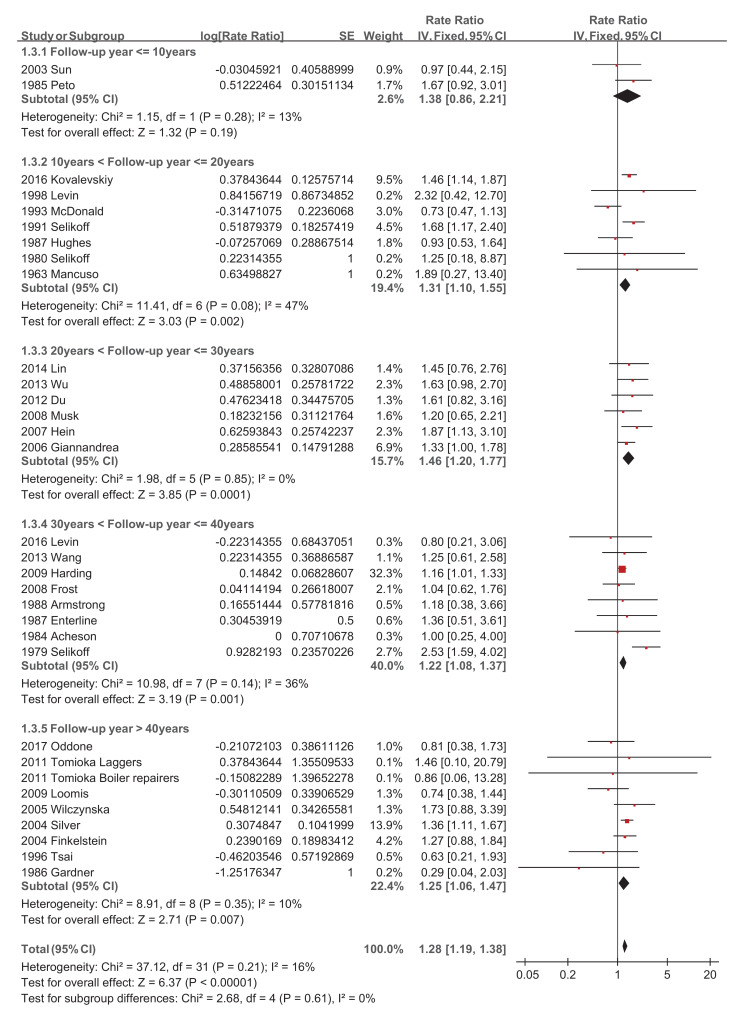
Subgroup analysis of SMRs of esophageal cancer based on follow-up years.

**Figure 6:**
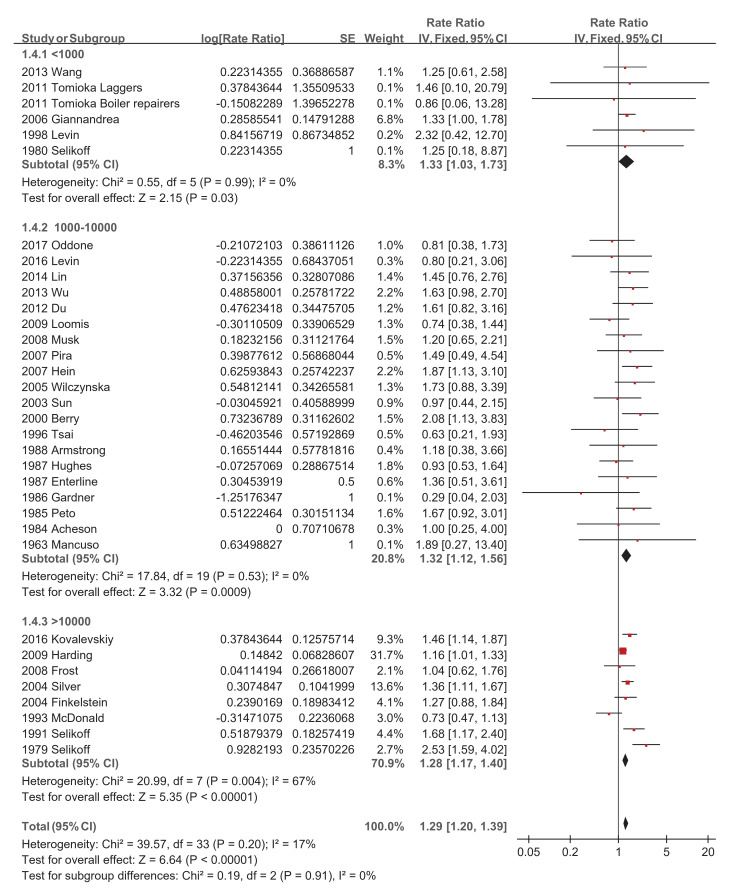
Subgroup analysis of SMRs of esophageal cancer based on sample size.

**Figure 7:**
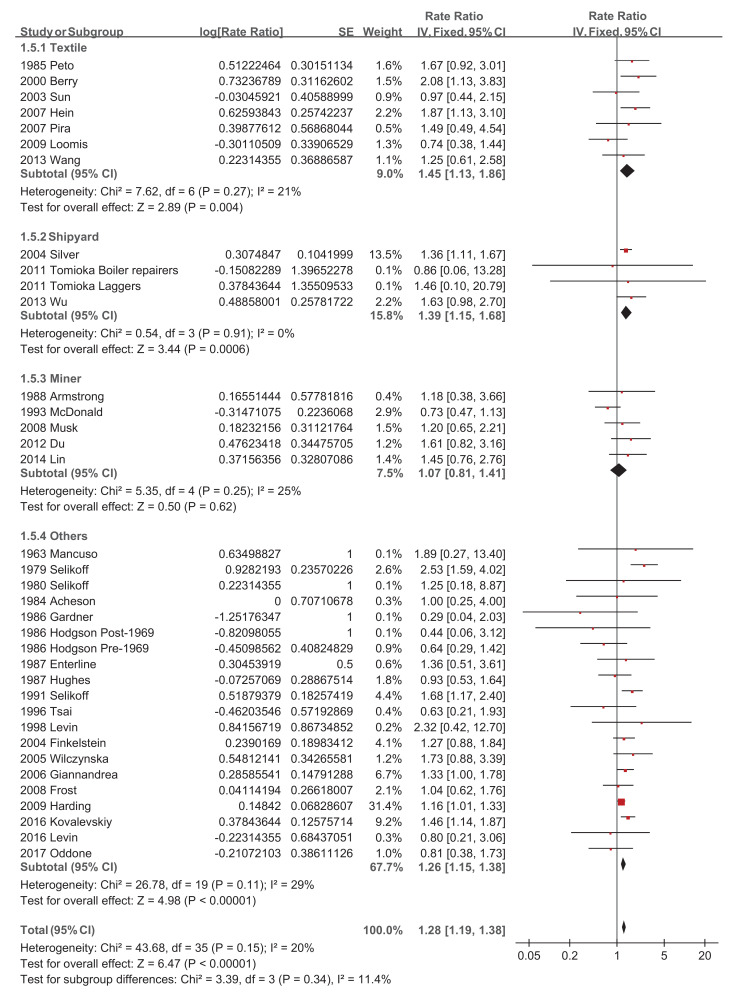
Subgroup analysis of SMRs of esophageal cancer based on industry classification.

**Figure 8:**
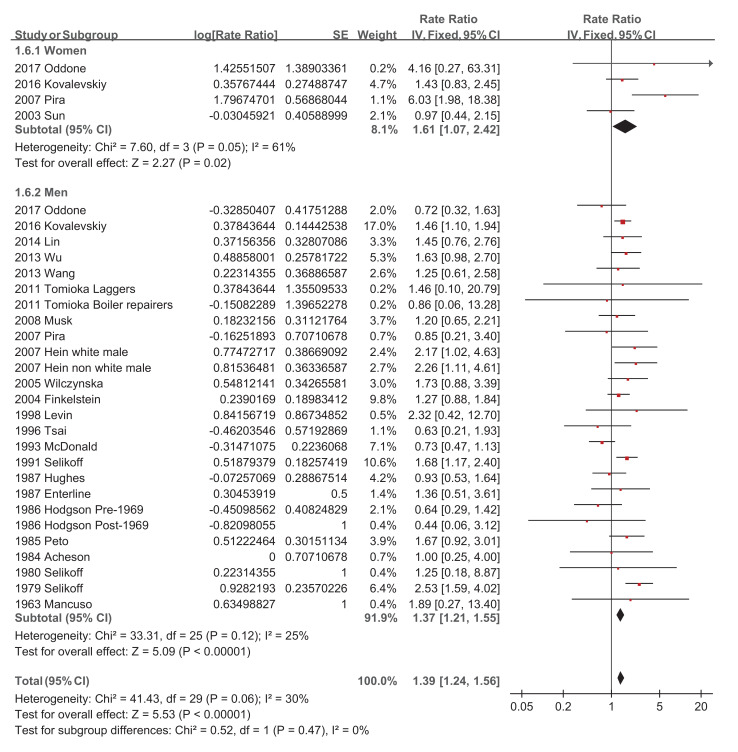
Subgroup analysis of SMRs of esophageal cancer based on sex.

**Figure 9:**
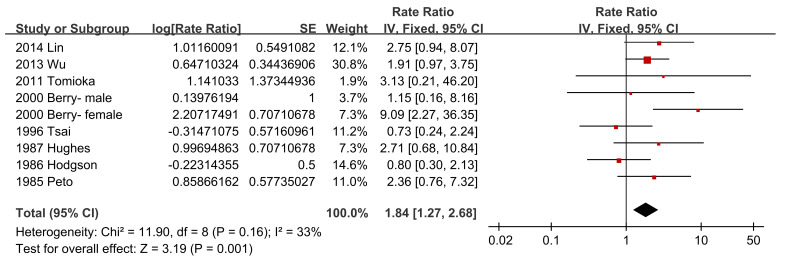
Subgroup analysis of SMRs of esophageal cancer in the highest asbestos exposure groups.

Regarding asbestos type, the chrysolite subgroup showed a significantly increased pooled SMR of 1.27 (95% CI 1.07–1.51, *p*-value = 0.006) [34,39,44,46,49,50], while amosite and crocidolite did not show significantly increased pooled SMRs of 1.14 (95% CI 0.53–2.47, *p*-value = 0.73) [20,22,32,51] and 1.20 (95% CI 0.70–2.05, *p*-value = 0.51) [28,42], respectively (Figure 4). Studies with a follow-up of no more than 10 years did not show a significantly increased pooled SMR of 1.38 (95% CI 0.86–2.21, *p*-value = 0.19) [23,34], while the other four subgroups showed significantly increased pooled SMRs, with 1.31 (95% CI 1.10–1.55, *p*-value = 0.002) [19,20,27,29,30,32,50], 1.46 (95% CI 1.20–1.77, *p*-value = 0.0001) [38,39,42,46,48,49], 1.22 (95% CI 1.08–1.37, *p*-value = 0.001) [21,22,26,28,41,43,47,51], and 1.25 (95% CI 1.06–1.47, *p*-value = 0.007) [24,31,35,36,37,44,45,52], respectively (Figure 5). The pooled SMRs of studies with participants <1000, 1000–10,000, and >10,000 were significantly increased by 1.33 (95% CI 1.03–1.73, *p*-value 0.03) [20,32,38,45,47], 1.32 (95% CI 1.12–1.56, *p*-value 0.0009) [19,22,23,24,26,27,28,31,33,37,39,40,42,44,46,48,49,51,52], and 1.28 (95% CI 1.17–1.40, *p*-value < 0.00001) [21,29,30,35,36,41,43,50], respectively (Figure 6). The pooled SMRs of the textile industry and shipyard significantly increased by 1.45 (95% CI 1.13–1.86, *p*-value = 0.004) [23,33,35,39,40,44,47] and 1.39 (95% CI 1.15–1.68, *p*-value = 0.0006) [36,45,48], respectively, while the pooled SMR of miners did not significantly increase at 1.07 (95% CI 0.81–1.41, *p*-value = 0.62) [28,30,42,46,49] (Figure 7). Both pooled SMRs of females and males were significantly increased, with 1.61 (95% CI 1.07–2.42, *p*-value = 0.02) [34,40,50,52] and 1.37 (95% CI 1.21–1.55, *p*-value < 0.00001) [19,20,21,22,23,25,26,27,29,30,31,32,35,37,39,40,42,45,47,48,49,50,52], respectively (Figure 8). The pooled SMR of eight studies in the highest asbestos exposure groups was significantly increased, 1.84 (95% CI 1.27–2.68, *p*-value = 0.001) [23,25,27,31,33,45,48,49] (Figure 9).

### 3.5. Risk of Bias Assessment

**Figure 10:**
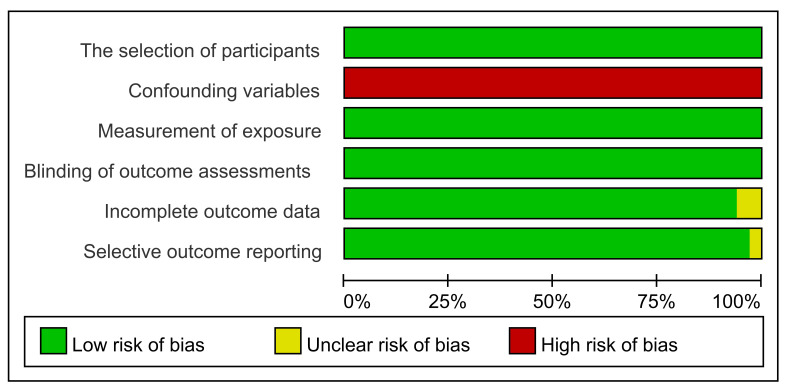
Graph of the Risk of Bias Assessment tool for Non-randomized Studies (RoBANS).

**Figure 11:**
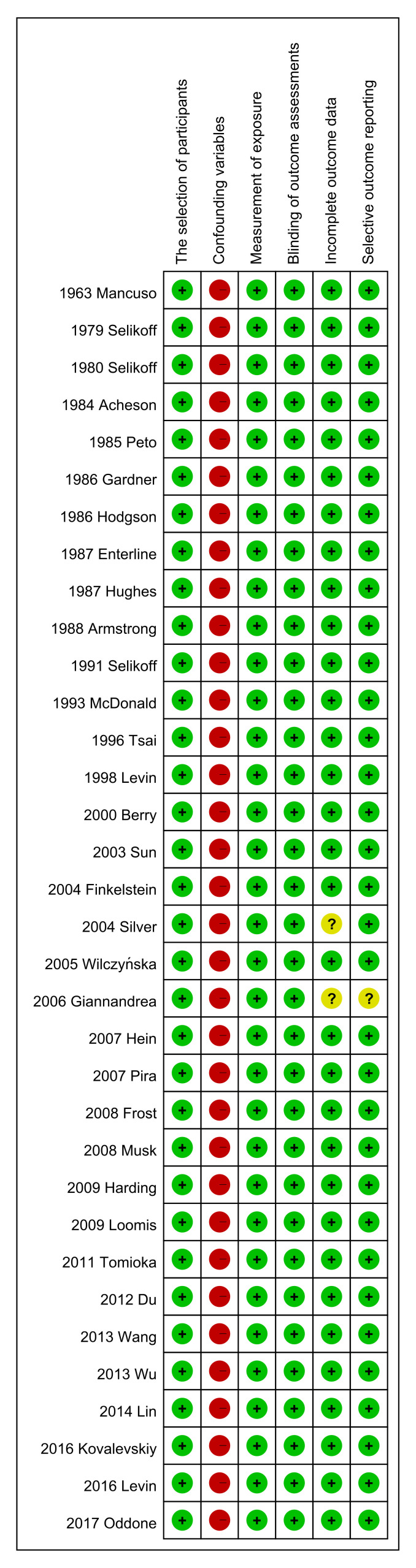
Graph summary of the Risk of Bias Assessment tool for Non-randomized Studies (RoBANS).

We evaluated the risk of bias of individual observational studies through the Risk of Bias Assessment tool for Non-Randomized Studies (RoBANS) [53]. The results are shown in Figure 10 and Figure 11, which revealed a low probability of bias except for three categories: (1) confounding variables, (2) incomplete outcome data and (3) selective outcome reporting. The probability of bias related to confounding factors was high risk in all studies due to unadjusted the potential confounding variables. The probability of bias related to incomplete outcome data was of unclear risk in 2006 Giannandrea et al. [38] and high risk in 2004 Silver et al. [36] The probability of bias related to selective outcome reporting was of unclear risk in an article from 2006 Giannandrea et al. [38].

## 4. Discussion

To our knowledge, this study is the most comprehensive and first investigation of 34 cohort studies on the SMR of esophageal cancer in asbestos-exposed participants. Previous investigators have conducted studies on the relationship between asbestos and esophageal cancer since the 1980s, but the evidence remains insufficient [19,24,25,29,30,33,36,39,44,45,50]. Based on description from Institute of Medicine (US) Committee on Asbestos in 2006, the evidence related to association between asbestos and esophageal cancer was insufficient [54]. In contrast, Li et al. had conducted a meta-analysis through 20 cohort studies and they found positive association between esophageal cancer and asbestos exposure [55]. However, we made a more comprehensive search from 1963 to 2017, and finally included 34 studies which generated 36 SMR for meta-analysis. In the meta-analysis study, we quantitatively assessed the relationship between asbestos exposure and esophageal cancer based on 34 cohort studies, and the results demonstrated an increased SMR (1.28) in esophageal cancer patients with occupational or environmental asbestos exposure; that is, participants with a history of asbestos exposure were 1.28 times more likely to die from esophageal cancer than the general population. Asbestos-related esophageal cancer may result from the generation of free radicals such as reactive oxygen species and chronic inflammation due to asbestos disposition [56]. According to an animal study from Møller et al., gastrointestinal tract exposure to asbestos increased the level of 8-oxo-7,8-dihydroguanine, causing oxidative-damaged DNA in the internal organs [57]. Nevertheless, more evidence is needed to clarify the mechanism of asbestos-induced esophageal malignancy.

Although all types of asbestos may be related to malignancy formation, different types of asbestos may have different potencies in inducing cancer. For example, amphibole fibers (including crocidolite and amosite) may cause a more harmful effect in inducing lung cancer than chrysolite fibers [1]. In our subgroup analysis of asbestos species, we found that exposure to chrysolite and mixed asbestos (containing chrysolite) was significantly associated with an increased SMR in esophageal cancer. A similar result was found in Wronkiewicz et al.’s study, which showed tissue surrounding pharyngeal cancer and laryngeal cancer in 6 cases with a history of occupational asbestos exposure through scanning electron microscopy, and chrysolite fibers were noted in the tissues of 3 cases [58]. Our results did not show a significantly increased SMR in participants who were exposed to amosite and crocidolite. Nevertheless, we found an elevated SMR or higher observed to expected death ratio of esophageal cancer after exposure to amosite and crocidolite in the included studies [20,28,32,42]. We believe that amosite and crocidolite also have the potential to induce esophageal cancer, but there are relatively few studies focusing on amosite and crocidolite. Chrysolite is currently the asbestos type that is most commonly used, so more studies discussing chrysolite and esophageal cancer were included in our study. More evidence is needed to clarify the relationship between asbestos type and esophageal cancer.

In the subgroup analysis of follow-up years, the results showed a significantly increased SMR of esophageal cancer in the four subgroups with more than ten years of follow-up. This is probably because of the long latency period of asbestos-related malignancy. Uguen et al. retrospectively reviewed and analyzed 146 patients with asbestos-related lung cancer, and the mean duration of the latency period was 10.5 ± 8.6 years [59]. According to a literature review published recently by Borrelli et al., the latency period of mesothelioma induced by occupational asbestos exposure was approximately 20–70 years [60]. Rarely could evidence discussing the duration of asbestos-related esophageal cancer be found. Based on our study, we recommend that workers with a history of occupational asbestos exposure should regularly be followed up for asbestos-related cancers, including esophageal cancer, for over 10 years.

In the subgroup analysis of industrial type, we found significant association between asbestos exposure and esophageal cancer in textile worker and shipyard, but not in asbestos miners. Our finding was lined with previous studies for asbestos-related cancer. Wang et al. observed chrysotile mining cohort and chrysotile textile worker for 26 years, and they found higher death risk of lung cancer in textile worker than in mine worker [61]. Based on measurement report from Berman, asbestos fibers are longer in textile industry dust than in mine dust, which may be a possible reason indicating workers in textile factory are more easily to get asbestos-related cancer than workers in mine [62]. Moreover, milled asbestos fibers (longer fiber asbestos) are also used in shipyard, which may be the possible reason that shipyard workers are in a higher risk of esophageal cancer. Nevertheless, relatively rare studies discussing the possible mechanism between asbestos industrial type and incidence of asbestos-related cancer. More studies are needed for further survey.

There are several limitations to our study. First, the SMR of esophageal cancer may be affected by the most common methodological challenges associated with attrition bias. For instance, workers in several studies did not work for over one year, which might indicate a health worker effect. However, this limitation is unavoidable, and we assessed the risk of bias for the 33 included studies with the RoBANS. Second, we made quantitative synthesis of those articles mentioning standardized mortality rate or providing observed group and expected group using meta-analysis. The results of the meta-analysis using publication that were not included due to their study design, such as case-control study design. This may have some selection bias and could be a limitation. Finally, the formation of esophageal cancer was affected by multiple factors despite the duration of asbestos exposure. Adjusting for possible confounders, including smoking or alcohol consumption, will provide more comprehensive and rigorous evidence, and this is a direction of future research.

## 5. Conclusions

In conclusion, the findings of this study indicated that occupational or environmental asbestos exposure might significantly increase the risk of esophageal cancer. In the subgroup analysis based on asbestos type, chrysolite was found to be significantly associated with esophageal cancer. Finally, due to the long latency period of asbestos-related esophageal cancer, we suggest that patients with a history of asbestos exposure should continue follow-up cancer screening for more than ten years.

## Figures and Tables

**Table 1:** Studies included in the meta-analysis study (N = 34).

Study (Year)	Country	N	Follow-up Period	Study Population	Asbestos type	Comparison	Observed/Expected of EC	SMR (95%CI)	Reference Population
1. Oddone (2017)	Italy	1818 males and females	1932–1992	asbestos-cement workers in the largest plant in Lombardy	Mixed asbestos	Overall Male Female	8/9.92 7/9.68 1/0.24	0.81 (0.35–1.59) 0.72 (0.29–1.49) 4.16 (0.10–23.16)	the National Institute of Health based on mortality
2. Kovalevskiy (2016)	Russian	16,596 males and females	1997–2010	Population-based	Chrysotile	Overall Male Female	66/45.21 51/34.93 15/10.49	1.46 (1.13–1.85) 1.46 (1.09–1.92) 1.43 (0.80–2.35)	Sverdlovsk resion
3. Levin (2016)	USA	1130 male and female	1979–2013	Tyler asbestos plant	Amosite	Overall	3/3.75	0.80 (0.16–2.34)	Life Table Analysis System, CDC, USA
4. Lin (2014)	China	1539 males	1981–2006	Chrysotile asbestos miners	Chrysolite	Overall (male)	9/6.22	1.45 (0.76–2.75)	Chinese national data
5. Wang (2013)	China	586 males and 279 females	1972–2008	Chrysolite textile factory	Primarily chrysotile	Male	7/5.59	1.25 (0.61–2.59)	Chinese nationwide data
6. Wu (2013)	Taiwan	4926 males and females	1985–2008	Ship breaks	Mixed asbestos	Male Male flame cutters Male lifters	17/10.4 13/5.8 4/2.2	1.63 (0.95–2.61) 2.24 (1.19–3.84) 1.82 (0.49–4.66)	General population of Taiwan
7. Du (2012)	China	1932 males and females	1981–2010	Chrysotile asbestos miners	Chrysolite	Overall	9/5.59	1.61 (0.73–2.82)	Chinese national death rate
8. Tomioka (2011)	Japan	90 male laggers159 male boiler repairers	1947–2007	Refitting shipyard	Mixed asbestos	Male laggers Male boiler repairers	1/0.68 1/1.16	1.46 (0.04–8.11) 0.86 (0.02–4.77)	Japanese male population
9. Harding (2009)	UK	98,117 males and females	1971–2006	British asbestos workers	Mixed asbestos	Overall	220/189.66	1.16 (1.01–1.32)	Great Britain
10. Loomis (2009)	USA	5770 males and females	1950–2003	Asbestos textile factories	Chrysolite	Overall	10/13.49	0.74 (0.36–1.36)	National population, USA
11. Frost (2008)	UK	31,302 males and females	1971–2005	Stripping/removal workers	Mixed asbestos	Overall	16/15.36	1.042 (0.596–1.692)	England, Wales and Scotlant
12. Musk (2008)	Australia	6943 males	1979–2001	Crocidolite mine	Crocidolite	Overall (male)	12/10	1.20 (0.62–2.10)	Western Australian male population
13. Hein (2007)	USA	3072 males and females	1979–2001	Asbestos textile plant	Chrysolite	Overall White males Nonwhite males	17/9.1 8/3.69 9/3.98	1.87 (1.09–2.99) 2.17 (0.94–4.28) 2.26 (1.03–4.28)	USA and South Carolina
14. Pira (2007)	Italy	1966 males and females	Up to 2004	Asbestos (mainly textile) company	Mixed asbestos	Overall Male Female	4/2.7 2/2.4 2/0.3	1.49 (0.41–3.81) 0.85 (NR) 6.03 (NR)	Italian National Institute of Statistics, and WHO
15. Giannandrea (2006)	Italy	427 deaths for gastrointestinal cancer	1980–2001	Population-based	Tremolite	Overall	NR	1.3309 (0.98–1.75)	Basilicata region
16. Wilczyńska (2005)	Poland	4497 males and females	1945–1999	Asbestos plant	Mixed asbestos	Male	10/5.78	1.73 (0.83–3.18)	General Poland population
17. Finkelstein (2004)	USA and Canada	25,285 males	1950–1999	Pipe trade workers	Not specified	Overall (male)	30/23.62	1.27 (0.86–1.81)	Ontario male population
18. Silver (2004)	USA	37,853 males and females	1952–1996	Portsmouth Naval Shipyard	Not specified	Overall	97/71.32	1.36 (1.11–1.67)	NIOSH personal computer Life Tabale Analysis System
19. Sun (2003)	China	5681 females	1990–2000	Manual spinning workers	Chrysotile	Overall (female)	6/6.18	0.97 (0.44–2.16)	Cixi City female population
20. Berry (2000)	UK	5100 males and females (12 cases)	Up to 1980	Textile and prefabricated cement pipes	Mixed asbestos	Overall	12/5.78	2.08 (0.44–2.16)	England and Wales
21. Levin (1998)	USA	753 males	1954–1972	Manufacture of asbestos pipe insulation materials	Amosite	Overall (male)	2/0.9	2.32 (0.28–8.39)	Empolys mortality and population data system, University of Pittsburgh, USA
22. Tsai (1996)	USA	2504 males	1948–1989	Refinery and petrochemical plant, Texas	Mixed asbestos	Overall (male)	4/6.4	0.63 (0.17–1.60)	Harris County, Texas
23. McDonald (1993)	Canada	11,000 males	1976–1988	Chrysotile miners and millers	Chrysotile	Overall (male)	20/27.39	0.73 (NR)	General population of Ouebec
24. Selikoff (1991)	USA and Canada	17,800 males	1967–1986	Asbestos insulation workers	Not specified	Overall (male)	30/17.80	1.68 (NR)	US National Center for Health Statistics
25. Armstrong (1988)	Australia	6916 males and females	1943–1980	Crocidolite mining and milling	Crocidolite	Overall	3/2.54	1.18 (0.38–3.66)	Western Australia
26. Enterline (1987)	USA	1074 males	1941–1980	Asbestos company	Not specified	Overall (male)	4/2.95	1.356 (NR)	US white men
27. Hughes (1987)	USA	6931 males	1970–1982	New Orleans asbestos cement plants	Primarily chrysotile	Overall (male)	12/12.9	0.93 (NR)	Louisiana mortality
28. Gardner (1986)	UK	2167 male and female	1941–1983	Asbestos cement factory	Mainly chrysotile, but some amosite	Overall	1/3.5	0.286 (NR)	England and Wales
29. Hodgson (1986)	UK	31,150 males	Up to 1981	British asbestos workers	Not specified	Pre-1969 Post-1969	6/9.4 1/2.3	0.637 (NR) 0.44 (NR)	England and Wales
30. Peto (1985)	UK	3211 males	1969–1973	Rochdale asbestos textile factory workers	Mainly chrysotile, but some crocidolite	Overall (male)	11/6.59	1.669 (NR)	England and Wales
31. Acheson (1984)	UK	5969 males	1947–1979	A manufacture factory	Amosite	Overall (male)	2/2	1.00 (NR)	England and Wales
32. Selikoff (1980)	USA	582 males	1961–1977	Amosite factory workers	Amosite	Overall (male)	1/0.8	1.25 (NR)	New Jersy white males
33. Selikoff (1979)	USA	17,800 males	1943–1976	US and Canada asbestos insulation workers	Not specified	Overall (male)	18/7.1	2.53 (NR)	US white male
34. Mancuso (1963)	USA	1495 males and females	1940–1960	An asbestos company	Not specified	Overall (male)	1/0.53	1.887 (NR)	Ohio State general population

CI: confidence interval; EC: esophageal cancer; NR: not reported; SMR: standardized mortality ratio.

## Data Availability

After this research is accepted and published, you can contact the author for the dataset.

## References

[1] IARC Working Group on the Evaluation of Carcinogenic Risks to Humans (2012). ASBESTOS (CHRYSOTILE, AMOSITE, CROCIDOLITE, TREMOLITE, ACTINOLITE AND ANTHOPHYLLITE). IARC Monographs on the Evaluation of Carcinogenic Risks to Humans. Arsenic, Metals, Fibres and Dusts.

[2] Albin M., Jakobsson K., Attewell R., Johansson L., Welinder H. (1990). Mortality and cancer morbidity in cohorts of asbestos cement workers and referents. Br. J. Ind. Med..

[3] Niklinski J., Niklinska W., Chyczewska E., Laudanski J., Naumnik W., Chyczewski L., Pluygers E. (2004). The epidemiology of asbestos-related diseases. Lung Cancer.

[4] Gaudino G., Xue J., Yang H. (2020). How asbestos and other fibers cause mesothelioma. Transl. Lung Cancer Res..

[5] Kratzke P., Kratzke R.A. (2018). Asbestos-Related Disease. J. Radiol. Nurs..

[6] Uhlenhopp D.J., Then E.O., Sunkara T., Gaduputi V. (2020). Epidemiology of esophageal cancer: Update in global trends, etiology and risk factors. Clin. J. Gastroenterol..

[7] Domper Arnal M.J., Ferrández Arenas Á., Lanas Arbeloa Á. (2015). Esophageal cancer: Risk factors, screening and endoscopic treatment in Western and Eastern countries. World J. Gastroenterol..

[8] Morgan R.W., Foliart D.E., Wong O. (1985). Asbestos and gastrointestinal cancer. A review of the literature. West. J. Med..

[9] Offermans N.S., Vermeulen R., Burdorf A., Goldbohm R.A., Keszei A.P., Peters S., Kauppinen T., Kromhout H., van den Brandt P.A. (2014). Occupational asbestos exposure and risk of esophageal, gastric and colorectal cancer in the prospective Netherlands Cohort Study. Int. J. Cancer.

[10] Wu W.T., Lin Y.J., Li C.Y., Tsai P.J., Yang C.Y., Liou S.H., Wu T.N. (2015). Cancer Attributable to Asbestos Exposure in Shipbreaking Workers: A Matched-Cohort Study. PLoS ONE.

[11] Clin B., Morlais F., Dubois B., Guizard A.V., Desoubeaux N., Marquignon M.F., Raffaelli C., Paris C., Galateau-Salle F., Launoy G. (2009). Occupational asbestos exposure and digestive cancers—A cohort study. Aliment. Pharmacol. Ther..

[12] Reid A., Ambrosini G., de Klerk N., Fritschi L., Musk B. (2004). Aerodigestive and gastrointestinal tract cancers and exposure to crocidolite (blue asbestos): Incidence and mortality among former crocidolite workers. Int. J. Cancer.

[13] De La Provôté S., Desoubeaux N., Paris C., Letourneux M., Raffaelli C., Galateau-Salle F., Gignoux M., Launoy G. (2002). Incidence of digestive cancers and occupational exposure to asbestos. Eur. J. Cancer Prev..

[14] Gustavsson P., Jakobsson R., Johansson H., Lewin F., Norell S., Rutkvist L.E. (1998). Occupational exposures and squamous cell carcinoma of the oral cavity, pharynx, larynx, and oesophagus: A case-control study in Sweden. Occup. Environ. Med..

[15] Parent M.E., Siemiatycki J., Fritschi L. (2000). Workplace exposures and oesophageal cancer. Occup. Environ. Med..

[16] Santibañez M., Vioque J., Alguacil J., Barber X., García de la Hera M., Kauppinen T. (2008). Occupational exposures and risk of oesophageal cancer by histological type: A case-control study in eastern Spain. Occup. Environ. Med..

[17] Rothman K.J., Greenland S., Lash T.L. (2008). Modern Epidemiology.

[18] Wang B. (2002). Estimation of Standardized Mortality Ratio in Epidemiological Studies. Master’s Thesis.

[19] Mancuso T.F., Coulter E.J. (1963). Methodology in Industrial Health Studies. Arch. Environ. Health Int. J..

[20] Selikoff I.J., Seidman H., Hammond E.C. (1980). Mortality effects of cigarette smoking among amosite asbestos factory workers. J. Natl. Cancer Inst..

[21] Selikoff I.J., Hammond E.C., Seidman H. (1979). Mortality experience of insulation workers in the United States and Canada, 1943—1976. Ann. NY Acad. Sci..

[22] Acheson E.D., Gardner M.J., Winter P.D., Bennett C. (1984). Cancer in a factory using amosite asbestos. Int. J. Epidemiol..

[23] Peto J., Doll R., Hermon C., Binns W., Clayton R., Goffe T. (1985). Relationship of mortality to measures of environmental asbestos pollution in an asbestos textile factory. Ann. Occup. Hyg..

[24] Gardner M.J., Winter P.D., Pannett B., Powell C.A. (1986). Follow up study of workers manufacturing chrysotile asbestos cement products. Br. J. Ind. Med..

[25] Hodgson J.T., Jones R.D. (1986). Mortality of asbestos workers in England and Wales 1971-81. Br. J. Ind. Med..

[26] Enterline P.E., Hartley J., Henderson V. (1987). Asbestos and cancer: A cohort followed up to death. Br. J. Ind. Med..

[27] Hughes J.M., Weill H., Hammad Y.Y. (1987). Mortality of workers employed in two asbestos cement manufacturing plants. Br. J. Ind. Med..

[28] Armstrong B.K., de Klerk N.H., Musk A.W., Hobbs M.S. (1988). Mortality in miners and millers of crocidolite in Western Australia. Br. J. Ind. Med..

[29] Selikoff I.J., Seidman H. (1991). Asbestos-associated deaths among insulation workers in the United States and Canada, 1967–1987. Ann. NY Acad. Sci..

[30] McDonald J.C., Liddell F.D., Dufresne A., McDonald A.D. (1993). The 1891–1920 birth cohort of Quebec chrysotile miners and millers: Mortality 1976-88. Br. J. Ind. Med..

[31] Tsai S.P., Waddell L.C., Gilstrap E.L., Ransdell J.D., Ross C.E. (1996). Mortality among maintenance employees potentially exposed to asbestos in a refinery and petrochemical plant. Am. J. Ind. Med..

[32] Levin J.L., McLarty J.W., Hurst G.A., Smith A.N., Frank A.L. (1998). Tyler asbestos workers: Mortality experience in a cohort exposed to amosite. Occup. Environ. Med..

[33] Berry G., Newhouse M.L., Wagner J.C. (2000). Mortality from all cancers of asbestos factory workers in east London 1933-80. Occup. Environ. Med..

[34] Sun T., Li L., Shi N., Zhang X. (2003). A 40-year cohort study on cancer mortality among female workers with manual spinning of chrysotile asbestos. Wei Sheng Yan Jiu.

[35] Finkelstein M.M., Verma D.K. (2004). A cohort study of mortality among Ontario pipe trades workers. Occup. Environ. Med..

[36] Silver S.R., Daniels R.D., Taulbee T.D., Zaebst D.D., Kinnes G.M., Couch J.R., Kubale T.L., Yiin J.H., Schubauer-Berigan M.K., Chen P.H. (2004). Differences in mortality by radiation monitoring status in an expanded cohort of Portsmouth Naval Shipyard workers. J. Occup. Environ. Med..

[37] Wilczyńska U., Szymczak W., Szeszenia-Dabrowska N. (2005). Mortality from malignant neoplasms among workers of an asbestos processing plant in Poland: Results of prolonged observation. Int. J. Occup. Med. Environ. Health.

[38] Giannandrea F., Binazzi A., Giordano F., Figà-Talamanca I. (2006). Exposure to amphibolic fibres and cancer of the gastrointestinal tract: An epidemiological survey in Lagonegro district (Southern Italy). Giornale Italiano di Medicina del Lavoro ed Ergonomia.

[39] Hein M.J., Stayner L.T., Lehman E., Dement J.M. (2007). Follow-up study of chrysotile textile workers: Cohort mortality and exposure-response. Occup. Environ. Med..

[40] Pira E., Pelucchi C., Piolatto P.G., Negri E., Discalzi G., La Vecchia C. (2007). First and subsequent asbestos exposures in relation to mesothelioma and lung cancer mortality. Br. J. Cancer.

[41] Frost G., Harding A.H., Darnton A., McElvenny D., Morgan D. (2008). Occupational exposure to asbestos and mortality among asbestos removal workers: A Poisson regression analysis. Br. J. Cancer.

[42] Musk A.W., de Klerk N.H., Reid A., Ambrosini G.L., Fritschi L., Olsen N.J., Merler E., Hobbs M.S., Berry G. (2008). Mortality of former crocidolite (blue asbestos) miners and millers at Wittenoom. Occup. Environ. Med..

[43] Harding A.H., Darnton A., Wegerdt J., McElvenny D. (2009). Mortality among British asbestos workers undergoing regular medical examinations (1971-2005). Occup. Environ. Med..

[44] Loomis D., Dement J.M., Wolf S.H., Richardson D.B. (2009). Lung cancer mortality and fibre exposures among North Carolina asbestos textile workers. Occup. Environ. Med..

[45] Tomioka K., Natori Y., Kumagai S., Kurumatani N. (2011). An updated historical cohort mortality study of workers exposed to asbestos in a refitting shipyard, 1947-2007. Int. Arch. Occup. Environ. Health.

[46] Du L., Wang X., Wang M., Lan Y. (2012). Analysis of mortality in chrysotile asbestos miners in China. J. Huazhong Univ. Sci. Technol. Med. Sci..

[47] Wang X., Lin S., Yu I., Qiu H., Lan Y., Yano E. (2013). Cause-specific mortality in a Chinese chrysotile textile worker cohort. Cancer Sci..

[48] Wu W.T., Lu Y.H., Lin Y.J., Yang Y.H., Shiue H.S., Hsu J.H., Li C.Y., Yang C.Y., Liou S.H., Wu T.N. (2013). Mortality among shipbreaking workers in Taiwan—A retrospective cohort study from 1985 to 2008. Am. J. Ind. Med..

[49] Lin S., Wang X., Yano E., Yu I., Lan Y., Courtice M.N., Christiani D.C. (2014). Exposure to chrysotile mining dust and digestive cancer mortality in a Chinese miner/miller cohort. Occup. Environ. Med..

[50] Kovalevskiy E.V., Schonfeld S.J., Feletto E., Moissonnier M., Kashanskiy S.V., Bukhtiyarov I.V., Schüz J. (2016). Comparison of mortality in Asbest city and the Sverdlovsk region in the Russian Federation: 1997–2010. Environ. Health.

[51] Levin J.L., Rouk A., Shepherd S., Hurst G.A., McLarty J.W. (2016). Tyler asbestos workers: A mortality update in a cohort exposed to amosite. J. Toxicol. Environ. Health B Crit. Rev..

[52] Oddone E., Ferrante D., Tunesi S., Magnani C. (2017). Mortality in asbestos cement workers in Pavia, Italy: A cohort study. Am. J. Ind. Med..

[53] Kim S.Y., Park J.E., Lee Y.J., Seo H.J., Sheen S.S., Hahn S., Jang B.H., Son H.J. (2013). Testing a tool for assessing the risk of bias for nonrandomized studies showed moderate reliability and promising validity. J. Clin. Epidemiol..

[54] Institute of Medicine Committee on Asbestos: Selected Health Effects (2006). The National Academies Collection: Reports funded by National Institutes of Health. Asbestos: Selected Cancers.

[55] Li B., Tang S.P., Wang K.Z. (2016). Esophagus cancer and occupational exposure to asbestos: Results from a meta-analysis of epidemiology studies. Dis. Esophagus.

[56] Peterson M.K., Mohar I., Lam T., Cook T.J., Engel A.M., Lynch H. (2019). Critical review of the evidence for a causal association between exposure to asbestos and esophageal cancer. Crit. Rev. Toxicol..

[57] Møller P., Danielsen P.H., Jantzen K., Roursgaard M., Loft S. (2013). Oxidatively damaged DNA in animals exposed to particles. Crit. Rev. Toxicol..

[58] Wronkiewicz S.K., Roggli V.L., Hinrichs B.H., Kendler A., Butler R.A., Christensen B.C., Marsit C.J., Nelson H.H., McClean M.D., Kelsey K.T. (2020). Chrysotile fibers in tissue adjacent to laryngeal squamous cell carcinoma in cases with a history of occupational asbestos exposure. Mod. Pathol. Off. J. US Can. Acad. Pathol..

[59] Uguen M., Dewitte J.D., Marcorelles P., Loddé B., Pougnet R., Saliou P., De Braekeleer M., Uguen A. (2017). Asbestos-related lung cancers: A retrospective clinical and pathological study. Mol. Clin. Oncol..

[60] Borrelli E.P., McGladrigan C.G. (2021). A Review of Pharmacologic Management in the Treatment of Mesothelioma. Curr. Treat. Options Oncol..

[61] Wang X., Lin S., Yano E., Yu I.T., Courtice M., Lan Y., Christiani D.C. (2014). Exposure-specific lung cancer risks in Chinese chrysotile textile workers and mining workers. Lung Cancer.

[62] Berman D.W. (2010). Comparing milled fiber, Quebec ore, and textile factory dust: Has another piece of the asbestos puzzle fallen into place?. Crit. Rev. Toxicol..

